# Effect of Sodium Sulfate Solution Coupled with Wetting–Drying Cycles on the Properties of Nano-Alumina-Modified Concrete

**DOI:** 10.3390/nano15020120

**Published:** 2025-01-15

**Authors:** Kai Gao, Dun Chen, Chunqing Li, Guoyu Li, Yuncheng Mao, Xuyang Wu, Anshuang Su, Hang Zhang, Xu Wang

**Affiliations:** 1 State Key Laboratory of Frozen Soil Engineering/Key Laboratory of Cryospheric Science and Frozen Soil Engineering, Northwest Institute of Eco-Environment and Resources, Chinese Academy of Sciences, Lanzhou 730000, China; gaokai@nieer.ac.cn (K.G.); guoyuli@lzb.ac.cn (G.L.); wuxuyang@nieer.ac.cn (X.W.); 2 University of Chinese Academy of Sciences, Beijing 100049, China; 3 International Research Center for China-Mongolia-Russia Cold and Arid Regions Environment and Engineering, Chinese Academy of Sciences, Lanzhou 730000, China; 4 Da Xing’anling Observation and Research Station of Frozen-Ground Engineering and Environment, Northwest Institute of Eco-Environment and Resources, Chinese Academy of Sciences, Jagdaqi 165000, China; 5 School of Civil Engineering, Northwest Minzu University, Lanzhou 730030, China; 182062710@xbmu.edu.cn; 6 Heilongjiang Provincial Hydraulic Research Institute, Harbin 150080, China; bridgecrete@163.com; 7 Electric Power Research Institute, State Grid Heilongjiang Electric Power Company Limited, Harbin 150030, China; zhanghang@hepri.hl.sgcc.com.cn; 8 Heilongjiang Transportation Information and Science Research Center, Harbin 150080, China; jtkj01@hljjtkj.com

**Keywords:** concrete, sodium sulfate, strength, microstructure, crystallization pressure, structure damage

## Abstract

The degradation of concrete caused by sulfate attack poses a significant challenge to its durability. Using nanomaterials to enhance the mechanical and durability properties of concrete is a promising solution. A study of the durability of nano-alumina (NA)-modified concrete by sulfate erosion was carried out. The results showed that the compressive strength, quality, and permeability of concrete to chloride ions decreased during its long-term erosion by a sodium sulfate solution. The NA-modified concrete exhibited higher resistance to erosion by the sodium sulfate than ordinary concrete (OPC), the rate of reduction in its tensile strength was low, and the resistance of sample NA1 to penetration by chloride ions decreased only by one-fifth compared with that of OPC. The results of mercury intrusion porosimetry (MIP) and scanning electron microscopy (SEM) tests showed that erosion had severely damaged the pore characteristics and micromorphology of concrete. The total porosities of the OPC and NA1 samples increased from 12.68% and 10.29% to 16.03% and 12.71%, respectively. Their microscopic morphology revealed loose particles and poor compactness. The leading causes of damage to concrete due to erosion by the sodium sulfate were its crystallization pressure and the swelling-induced stress caused by the deposition of crystals in its pores. This study demonstrates that NA can significantly enhance the durability of concrete against sulfate attack, offering valuable insights for strategic applications of NA in concrete materials.

## 1. Introduction

Concrete is widely used in construction because of its unique physical and mechanical properties. In certain environments, however, it is often eroded by inorganic salts, especially sulfates, such that its durability significantly decreases. Sulfate attack on cementitious materials is a complex chemo-mechanical process, encompassing sulfate diffusion, crystallization growth, and microcrack growth [1]. As the ambient temperature and humidity change, the sulfate solution penetrates into the pores of the concrete and undergoes a chemical reaction to generate crystallization pressure that damages the specimen and can lead to structural failure [2,3].

Calcium hydroxide (CH) and calcium silicate hydrate (C-S-H) are the main targets of sulfate attacks in concrete, the properties of which deteriorate due to the dehydration of calcium hydroxide and the decomposition of calcium silicate hydrate gel. The ettringite crystals formed by erosion by the sulfate grow and expand. This, coupled with the low tensile strength of concrete, leads to swelling-induced stress caused by erosion that is often higher than the tensile strength of concrete and leads to cracking and softening. Cracking increases the permeability of concrete, thereby accelerating the deterioration in the performance of concrete materials [4]. When the concentration of sulfate is high, gypsum crystals are formed, in addition to the aluminite crystals, through an ion exchange reaction during erosion. The gypsum crystals produced by the sulfate reaction also lead to a smaller expansion, which can lead to damage through a reduction in the stiffness, strength, and adhesion of concrete.

Sodium sulfate is the most widely used solution in tests of sulfate-induced corrosion, including surveys on the weathering of natural rock and durability tests of building materials. The porous material is usually soaked in a sodium sulfate solution in the test, and it is removed and dried at a specific temperature to form anhydrous sodium sulfate. Once it is subsequently cooled to room temperature, the sample is soaked in the sodium sulfate solution once again to hydrate it. Sodium sulfate is highly destructive when this process is repeated several times, where most of the damage caused by it occurs during the immersion phase [5]. This damage has been mainly attributed to the volumetric expansion caused by the hydration reaction [6]. It results from the growth of miscanthus crystals in a highly supersaturated solution produced by the dissolution of sodium sulfate [7,8]. The results of evaporation experiments by Rodriguez-Navarro and Doehned [9,10] showed that a saturated sodium sulfate solution caused much more damage to limestone than the evaporation of a saturated sodium chloride solution. Theoretically, the crystallization pressure generated by confined crystals increases with the degree of supersaturation of the solution [11]. Experiments have also shown that the growth of anhydrous sodium sulfate and miscanthus nitrate crystals may cause damage, depending on the relative humidity. When the relative humidity is lower than 50%, anhydrous sodium sulfate is better formed, while sodium sulfate decahydrate is more abundant when the relative humidity is greater than 50%. Experiments by Yang et al. [12] and Wang [13] showed that sodium sulfate can react with calcium hydroxide to produce gypsum, which causes concrete to swell and crack. Tian and Cohen [14] demonstrated that the swelling of ettringite and gypsum, caused by the reaction of sodium sulfate, can lead to the failure of concrete. Equations derived by Steiger and Asmussen [15] showed that the crystallization pressure is obtained mainly due to gypsum and ettringite.

Many studies have shown that adding nanomaterials to concrete can improve its physical and mechanical properties and durability [16,17]. Most such research has focused on nano-SiO2 [18,19,20,21,22], while few studies have examined the durability of NA-modified concrete. Although studies have shown that NA can improve the physical and mechanical properties of concrete [23,24], whether it can enable concrete to resist erosion by sulfates remains to be verified. In this paper, we investigate the use of nano-NA to improve the performance of concrete based on experiments under the W–D cycles of erosion in a sodium sulfate solution and discuss the adverse effects of erosion on the performance of concrete at the macroscopic and microscopic scales. We theoretically analyze the mechanism of the damage caused to concrete by sodium sulfate. The results provide a reference for construction projects that involve the use of concrete in an environment containing sulfates and are important for better understanding the process of failure of concrete under the action of W–D cycles using a sodium sulfate solution.

## 2. Experiments

### 2.1. Raw Materials

**Figure 1:**
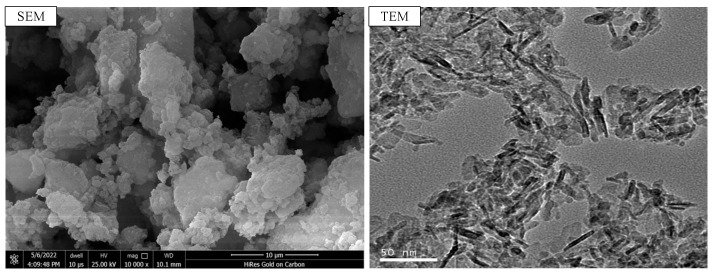
SEM and TEM images of nano-Al_2_O_3_.

The cement utilized in the test was P.O 42.5 ordinary Portland cement, produced by Gansu Qilianshan Cement Group Co., Ltd. in Lanzhou, China, and its chemical components are shown in Table 1, conforming to the Chinese standard GB175–2007 [25]. Both river sands and crushed stones were produced in Baiyin City, Gansu Province, and their strength, particle size, and gradation satisfied the Chinese standard JGJ 52–2006 [26]. The river sands had a particle size smaller than 4.75 mm, an apparent density of 2.69 g/cm^3^, and a mud content of 1.0%. The crushed stones had a size range of 5–20 mm, an apparent density of 2.85 g/cm^3^, and a mud content of 0.6%. A polycarboxylate superplasticizer with a water-reducing rate of 25% was utilized. Nano-Al_2_O_3_ (NA) is a crystalline substance with a particle diameter of less than 20 nm, produced by Suzhou Yuante New Material Co., Ltd. in Suzhou, China, which is used to replace a small amount of cement in concrete. Table 2 shows the physical properties of NA; Figure 1 shows the morphology of NA.

### 2.2. Mix Proportion

Table 3 shows the mixed proportion of concrete, where the ratio of water to binder (w/b) is 0.4. In the design of the concrete mix ratio, the method of calculating the amount of coarse and fine aggregates is the quality method. NA replaces 0%, 1%, 2%, and 3% of cement, respectively, and the corresponding prepared specimens are OPC, NA1, NA2, and NA3. OPC is the control specimen, and the amount of water is reduced to 1% of the weight of the cementitious materials. The mixed proportion of the cement mortar is the same as that of concrete, except that the aggregate is removed by filtering from the concrete mixture.

### 2.3. Sample Preparation

**Figure 2:**
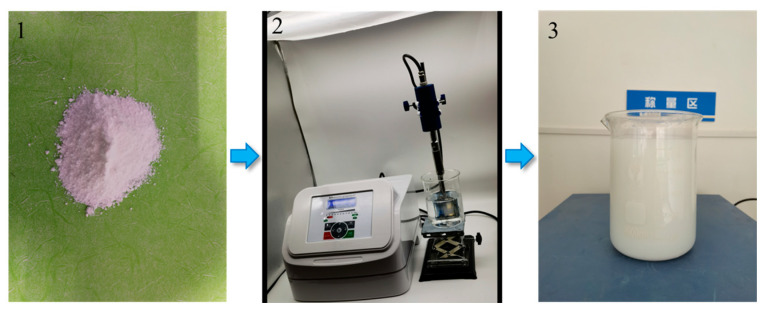
Preparation process of the suspension.

The suspension is prepared by first pouring the water reducer into the water and stirring for 10 min to obtain the aqueous solution, pouring NA into the aqueous solution, and dispersing it with an ultrasonic processor for 30 min, which obtains the suspension used in the test. The dispersion process is shown in Figure 2, which shows (1) nano-alumina, (2) ultrasonic dispersion, and (3) dispersed suspension. The concrete mixture is prepared by first pouring the cement, sand, and aggregate into the mixer and mixing evenly, and then the suspension is poured into the mixture and is stirred for 1–2 min, and the concrete mixture used in the test is obtained. The specimens are prepared as follows. The concrete samples are made into sizes of 100 × 100 × 100 mm^3^ and Φ100 × 50 mm^3^ for the compressive strength and Coulomb electric flux tests, and the cement mortar samples are made into sizes of Φ50 × 100 mm^3^ for the mercury intrusion porosimeter test. Finally, for the curing samples, all specimens were demolded after 12 h and then placed in a standard curing room (20 ± 2 °C) for 28 days.

### 2.4. Testing Methods

#### 2.4.1. W–D Cycle Test

**Figure 3:**
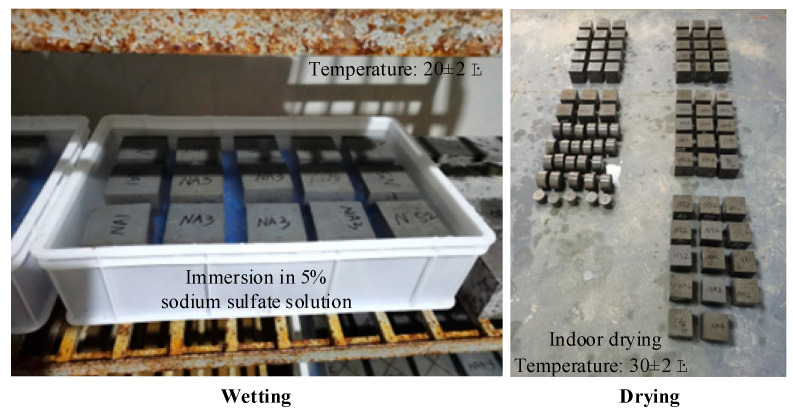
Sodium sulfate solution and W-D cycle coupling.

The W–D cycle test of a sample subjected to a sodium sulfate solution is shown in Figure 3. The specimen was first soaked in the sodium sulfate solution in the turnover box of a standard curing room and was then dried in a working room with a constant temperature. A W–D cycle lasted one week, with five days of wetting and two days of drying. The liquid used for immersion was a sodium sulfate solution with a concentration of 5% that was replaced once a month. To ensure that the sample was in complete contact with the sodium sulfate solution in the wetting stage, a hollow grid liner was placed at the bottom of the turnover box. The spacing between the samples was 2 cm, and the sodium sulfate solution was 2 cm higher than the top surface of the sample. The soaked samples were taken out of the turnover box in the drying stage and placed in the workshop to dry. The temperature was controlled at 30 ± 2 °C.

#### 2.4.2. Test of Macroscopic Characteristics

**Figure 4:**
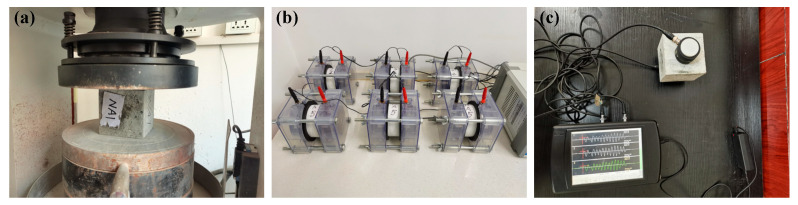
(**a**) Compressive strength; (**b**) Coulomb electric flux; (**c**) ultrasonic wave velocity.

Figure 4 shows the tests conducted to measure the compressive strength, Coulomb electric flux, mass loss, and velocity of ultrasonic waves of the concrete samples during the W–D cycle according to the Chinese standards [27,28]. The loading rate was 0.5 MPa/s, the constant DC voltage was 60 ± 0.1 V, the accuracy of mass was 0.5 g, and the frequency of the waves was 50 KHz.

#### 2.4.3. Microstructure Test

**Figure 5:**
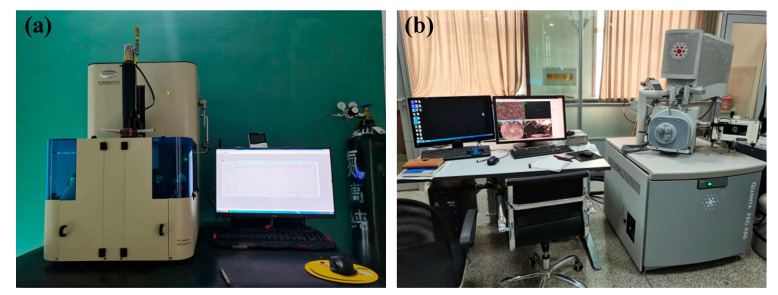
(**a**) The MIP test; (**b**) the SEM test.

Figure 5 shows the MIP and SEM tests of the specimens. Samples with dimensions of Φ8 × 10 mm^3^ were extracted from mortar samples using an electric drill for the MIP test. They were placed in ethanol to prevent a hydration reaction before the test. The SEM samples were taken from crushed fragments of the concrete specimens used for the test of compressive strength. The samples were dried at ambient temperature, placed on a paste–aggregate interface, sprayed with gold, and scanned using SEM to observe their microstructure.

## 3. Results and Discussion

### 3.1. Physical and Mechanical Properties

#### 3.1.1. Compressive Strength

**Figure 6:**
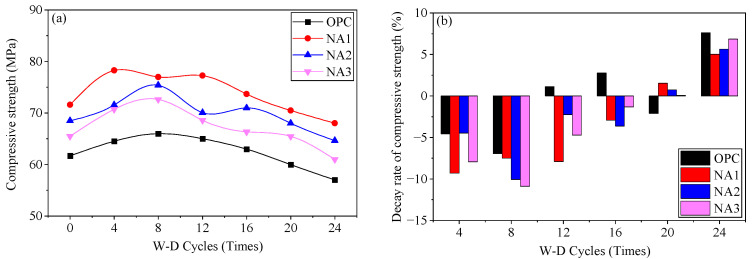
The compressive strength (**a**) and compressive strength decay rate (**b**) of the specimen during the W-D cycle.

Previous studies have shown that the compressive strength of NA-modified concrete is higher than that of ordinary concrete and decreases with an increase in the amount of NA. The compressive strengths of the samples were NA1, NA2, NA3, and OPC in descending order [29,30]. A sulfate attack is a slow and continuous process, during which structural degradation becomes progressively more pronounced as salt intrusion increases. The changes in the compressive strength of the specimens with the number of W–D cycles are shown in Figure 6a. The compressive strength of all specimens increased first and then decreased, where the eighth cycle was the cut-off point. Their compressive strength decreased due to the crystallization pressure generated by the crystallization of the crystals formed by the reaction of sodium sulfate and the dry shrinkage-induced deformation caused by the W–D cycle. The compressive strength of the specimen did not decrease at the beginning but only gradually after eight W–D cycles. This was observed because the crystallization pressure and dry shrinkage-induced deformation developed slowly, and their destructive effects were subjected to the W–D cycles, such that their compressive strength had not yet reached its maximum value. It gradually increased and significantly exceeded the erosive and destructive effects of the sodium sulfate solution in the first few cycles. After eight cycles, the crystallization pressure generated by the sodium sulfate crystals and the dry shrinkage-induced deformation caused by W–D cycles became dominant and had noticeable adverse effects on the mechanical properties of concrete. Its compressive strength began to decrease with an increasing number of W–D cycles.

Figure 6b shows the rate of reduction in the compressive strength of the specimens. It did not decrease in the initial W–D cycles but increased instead. The compressive strength of ordinary concrete decreased sooner than that of concrete with NA. The compressive strength of the OPC specimen began to decrease after the 12th cycle, while that of specimens NA1, NA2, and NA3 did so only after the 20th cycle. The reduction in the compressive strengths of all specimens was evident after the 24th W–D cycle, with the highest rate of reduction recorded by OPC and the lowest by NA1, followed by NA2 and NA3. These results show that incorporating NA in concrete can mitigate the reduction in its compressive strength by the sodium sulfate solution. It had a higher resistance to corrosion than ordinary concrete, and the optimal amount of NA in concrete was determined to be 1%. The erosion of concrete by the sodium sulfate solution is a long process, and its effect only gradually becomes apparent.

#### 3.1.2. Charge Passed

**Figure 7:**
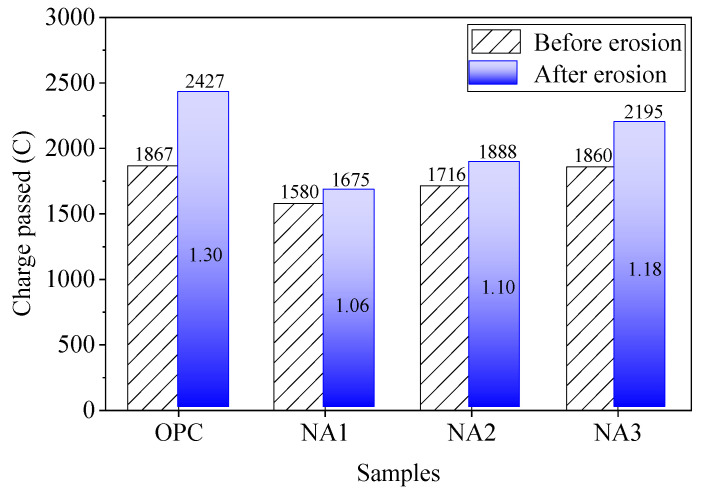
Charge-passed value of the specimen before and after erosion.

The charge-passed values of concrete were tested before and after the W–D cycles, and the difference was used to evaluate the permeability of the samples. The magnitude of the charge-passed value can be used to measure the strength of the resistance of concrete to penetration by chloride ions. The two are negatively correlated. The smaller the charge-passed value is, the stronger the resistance of concrete to permeation by chloride ions, and vice versa. Figure 7 shows that the charge-passed values of OPC, NA1, NA2, and NA3 were 1867, 1580, 1716, and 1860 C, respectively, before being subjected to sodium sulfate. After erosion by sodium sulfate, the charge-passed values of OPC, NA1, NA2, and NA3 were 2427, 1675, 1888, and 2195 C, respectively. These results show that the charge-passed value of the concrete samples increased after erosion by sodium sulfate, which means that their resistance to penetration by chloride ions weakened.

The resistance of concrete to permeation by chloride increased with the addition of NA. However, the samples weakened with the increase in NA content, mainly due to the adsorption of the NA particles. They adsorbed free water during the hydration reaction, and this led to poor properties of the concrete mixture and a large number of capillary pores during the formation process of cement stone. The rate of the growth of the charge-passed value of OPC was 30% after the W–D cycles, while the rates of growth of specimens NA1, NA2, and NA3 were 6%, 10%, and 18%, respectively. Compared with the other samples, the charge-passed value of NA1 decreased by only 6% after erosion by the sodium sulfate solution, which was one-fifth of the loss in the value of the OPC specimens. This indicates that NA1 had good osmotic resistance, even when subjected to a sulfate attack. Therefore, we recommend using concrete modified with 1% of NA when concrete is used in projects in an environment containing sodium sulfate.

#### 3.1.3. Mass Loss

Before implementing the W–D cycles, we selected a group of marked samples, soaked them in tap water, and weighed them after 48 h. The measured weights were used as their initial masses. We then carried out the W–D cycle tests of the samples as they were eroded by sodium sulfate. We then weighed the specimens once again while considering four W–D cycles as a single cycle to assess the loss of mass. The rate of mass loss of the specimen was calculated as follows:

(1) $$W=\frac{M_{0}-M_{N}}{M_{0}}$$

where W is the mass loss rate of the concrete sample, ‰; $M_{0}$ is the mass of the concrete sample before the W–D cycles, g; and $M_{N}$ is the mass of the concrete sample after N cycles, g.

**Figure 8:**
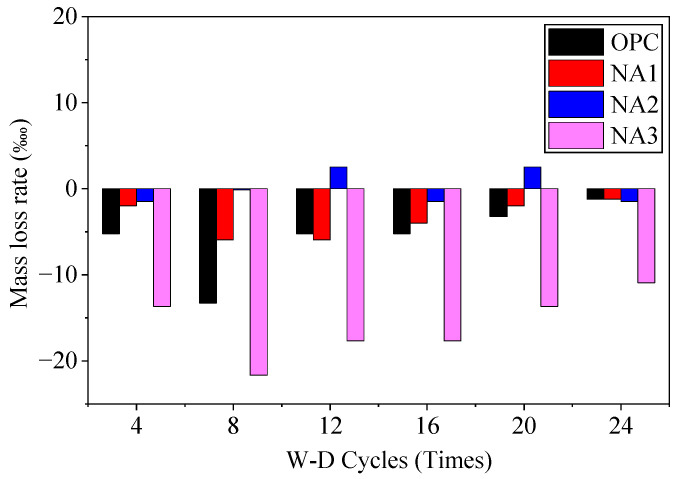
The mass loss rate of the specimen.

Figure 8 shows that specimens OPC, NA1, NA2, and NA3 recorded nearly no mass loss during the W–D cycle, and their masses increased instead. The masses of specimens OPC and NA3 increased particularly significantly. This, combined with the fact that the surface of the sample did not peel off during the W-D cycles, shows that the sodium sulfate solution did not cause noticeable damage to the surface of concrete during the W–D cycles. However, sodium sulfate crystals filled the pores inside the concrete to increase their mass. Their mass first increased and then decreased, indicating that the crystals had caused some damage to concrete during their circulation through it. The mass of sample NA3 changed because it contained too much NA. Its surface thus adsorbed a large amount of free water, leading to a decline in the workability of concrete through the formation of more internal pores as it is poured. The results indirectly show that sulfate-induced erosion and destruction occurred mainly due to the crystallization pressure, which was initiated in the pores of concrete and required a long erosion time. It was thus unfeasible to evaluate the resistance of concrete to erosion by sulfate based on the loss in its mass over a short period.

#### 3.1.4. Velocity of Ultrasonic Waves

**Figure 9:**
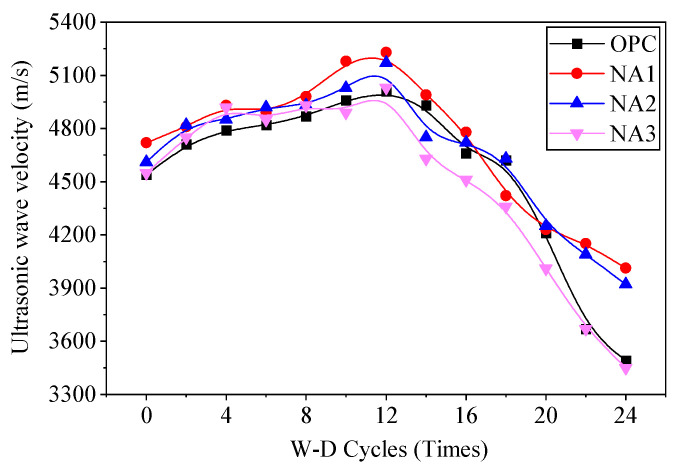
The relationship between ultrasonic velocity and W-D cycles.

The velocity of ultrasonic waves through the concrete samples changed during their erosion by the sodium sulfate solution in the W–D cycles, as shown in Figure 9. The velocity of ultrasonic waves in all samples began to decrease during the 12 W–D cycle before, and then it increased slowly with the number of W–D cycles. The velocity of ultrasonic waves in specimen NA1 was the highest, while that in specimen OPC was the lowest. The velocity of ultrasonic waves in specimen NA2 was lower than that in NA3. After the 12th cycle, the velocity of ultrasonic waves of the specimens decreased rapidly with a growing number of W–D cycles. We can infer from the results that the structure of the concrete was intact and was not damaged by the sodium sulfate solution under fewer than 12 W–D cycles for two reasons. On the one hand, although concrete was eroded by the sodium sulfate solution after 28 days of aging, its physical properties became more robust over time as well, and the enhancement in its overall performance was greater than the effect of its erosion by sodium sulfate. On the other hand, the crystals generated in the sodium sulfate solution under short-term erosion filled the pores of the concrete to make its internal structure denser, and this led to a slight increase in the velocity of ultrasonic waves. After 12 W–D cycles, the physical properties of concrete did not further develop, and the erosive effect of sodium sulfate became more apparent. Pores and cracks began to appear in the internal structure of concrete, the aggregates and mortar were no longer tightly bonded to each other, and the shape of the structure loosened. The looseness of the concrete structure became increasingly evident with an increase in the number of W–D cycles, and because of this, the velocity of ultrasonic waves of the specimens decreased. As in the previous tests, specimen NA1 showed the highest resistance to erosion by the sodium sulfate solution.

In general, the physical and mechanical properties of the concrete samples were affected due to erosion by the sodium sulfate solution during the W–D cycles. Concrete was significantly damaged only after long-term erosion, while short-term erosion had little adverse effect on its properties. NA-modified concrete with 1% NA had a stronger resistance to sulfate-induced erosion than ordinary concrete.

### 3.2. Microstructure

#### 3.2.1. Porosity

To analyze the effects of the sodium sulfate solution on the characteristics of pores of concrete during the W–D cycles, we conducted MIP tests on the mortar samples OPC and NA1 before and after erosion. OPC and NA1 represent the pre-erosion specimens, while OPC* and NA1* represent the post-erosion specimens.

**Figure 10:**
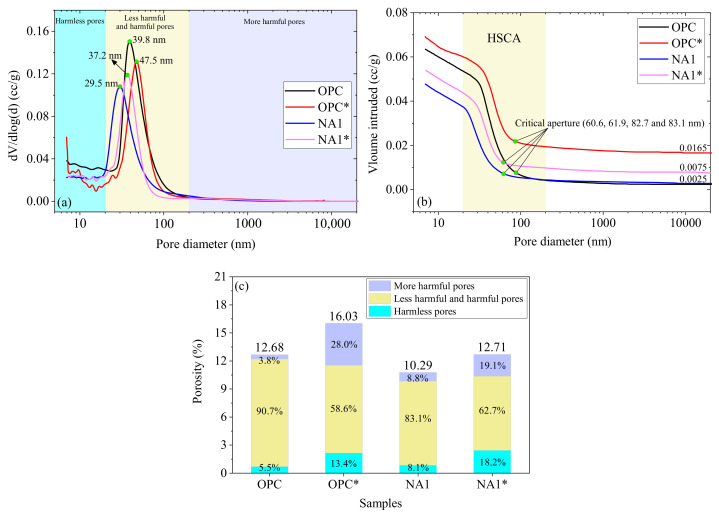
Porosity character of the cement mortar samples: (**a**) the pore size distribution; (**b**) the pore distribution under cumulative intrusion; (**c**) porosity.

As shown in Figure 10a, the pore size distributions of the mortar samples OPC, OPC*, NA1, and NA1* were obtained by the MIP test and were found to be mainly distributed between 7 and 20,000 nm. We divided the pores in cement mortar according to the effect of their size on the performance into harmless pores (<20 nm), less harmful pores (20–50 nm), harmful pores (50–200 nm), and more harmful pores (>200 nm) [31,32,33]. The figure shows that pores with diameters smaller than 200 nm in specimens OPC and NA1 mainly changed before and after their erosion by sodium sulfate, especially with respect to the number of less harmful and harmful pores. Peaks of waves were observed on the differential curves and corresponded to the most probable apertures of the mortar specimens. The most probable apertures of specimens OPC, OPC*, NA1, and NA1* were 39.8, 47.5, 37.2, and 29.5 nm, respectively, in an ascending order of magnitude of NA1 < NA1* < OPC < OPC*. This shows that the most probable apertures of specimen NA1 were smaller than those of OPC, while those of both NA1 and OPC increased after undergoing W-D cyclic erosion with the sodium sulfate solution. In other words, corrosion by sodium sulfate led to new harmful pores in concrete.

Figure 10b shows the cumulative curves of intrusion by mercury into the mortar specimens in the MIP test. The high slope coefficient area (HSCA) of the curves was mainly distributed in the range of 20–200 nm, indicating that the specimens were dominated by pores with diameters between 20 and 200 nm. The critical aperture of specimens OPC, OPC*, NA1, and NA1* were 83.1, 82.7, 61.9, and 60.6 nm, respectively, indicating that the erosion barely changed their critical aperture. The intrusion of mercury into specimens containing pores with diameters in the range of 200–20,000 nm was nearly unchanged, indicating that there were nearly no pores within this range of diameters in the specimen. The corresponding values of intrusion by mercury into specimens OPC, OPC*, NA1, and NA1* when they contained pores with a diameter of 20,000 nm were 0.0025, 0.0165, 0.0025, and 0.0075 cc/g, respectively. The values of mercury intrusion of OPC* and NA1* were larger than those of OPC and NA1. The largest value was obtained for OPC*, followed by NA1*. This suggests that the surface structures of OPC* and NA1* were rougher than those of OPC and NA1, and the surface of the former was the roughest due to erosion by the sodium sulfate solution, such that small voids in the sample became larger.

Figure 10c shows that the total porosities of OPC, OPC*, NA1, and NA1* were 12.68%, 16.03%, 10.29%, and 12.71%, respectively. The incorporation of NA reduced the total porosity of the mortar, while erosion by sodium sulfate increased it. NA thus had a positive effect on the samples, while erosion had an adverse effect. The ratios of various types of pores in the sample remained changed. Following erosion by sodium sulfate, the ratios of harmless and harmful pores decreased, while those of less harmful pores and more harmful pores decreased. For example, the ratios of less harmful and harmful pores decreased from 90.7% to 58.6% in OPC and from 83.1% to 62.7% in NA1. The influence of harmless pores on the performance of concrete was minimal and can thus be ignored. The more harmful pores are formed in it as it is poured, and they have a significant impact on its performance. This can, however, be avoided by following the standard requirements of construction. Less harmful and harmful pores are formed during the hydration of cement, which is inevitable, but the incorporation of NA can improve its performance in this regard. The results show that in case of erosion by the sodium sulfate solution, more pores were generated inside the concrete, while small pores developed into larger ones to increase its total porosity and affect its performance.

#### 3.2.2. Microstructural Morphology

**Figure 11:**
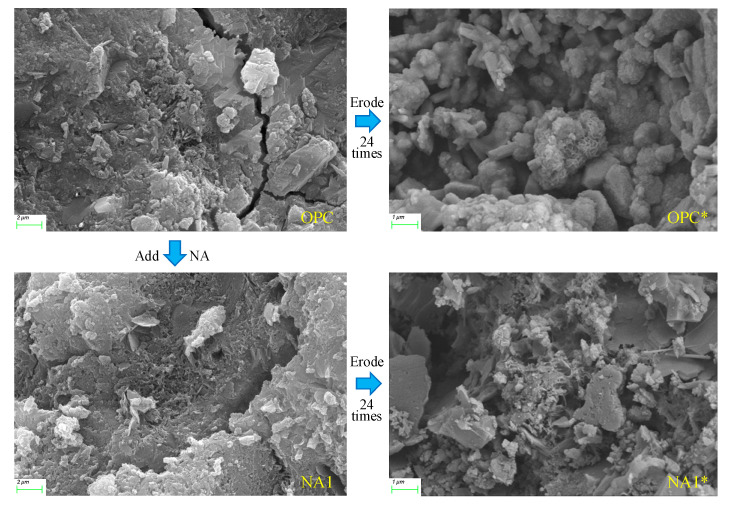
The SEM micrographs of OPC, NA1, OPC*, and NA1* samples.

We chose pressed fragments of concrete specimens OPC, OPC*, NA1, and NA1* for SEM tests to observe their microstructural morphology. Figure 11 shows that features of the structural surface features, such as particle morphology, cracks, and pores, were apparent in all specimens, and cracks and pores were almost always present. Before they were eroded by the sodium sulfate solution, the structural surfaces of specimens OPC and NA1 were well integrated, with sparse pores and angular granularity. After erosion, the structure of specimen OPC became loose, the number of pores in it increased, and the angularity of the granules disappeared; it was chemically corroded. The number of pores in specimen NA1 increased slightly, but the signs of the granules being chemically corroded were not prominent. These results show that erosion by sodium sulfate caused significant damage to the internal structure of concrete, especially ordinary concrete. The crystalline matter was severely chemically corroded, the number of pores increased in number and size, and its internal structure loosened. Compared with ordinary concrete, erosion by sodium sulfate caused less damage to the internal structure of NA-modified concrete. This indicates that incorporating NA into concrete enhances its resistance to erosion by sulfate.

### 3.3. Effect of Erosion

#### 3.3.1. Chemical Reaction

**Figure 12:**
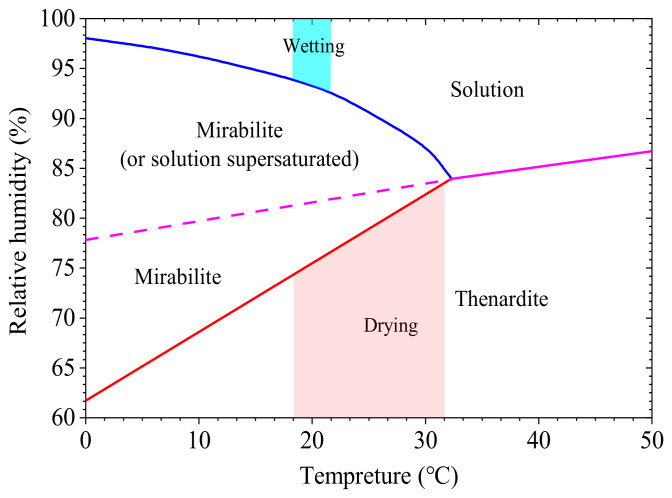
Phase diagram for sodium sulfate [8].

Concrete undergoes W–D cycles in a sodium sulfate solution to produce various crystals. Sodium sulfate crystals are continuously precipitated with changes in the temperature and humidity during the W–D cycles, as shown in Figure 12. Moreover, the sodium sulfate solution penetrates into the internal pores of concrete and reacts with substances around them to form ettringite and gypsum crystals. Specifically, sodium sulfate reacts with calcium hydroxide to produce gypsum and with calcium silicate hydrate to form gypsum, which, in turn, results in gypsum reacting with calcium aluminate hydrate to form ettringite crystals [34,35,36]. The reaction chemistry is as follows:

(2) $${Na}_{2}{SO}_{4}+Ca{\left(OH\right)}_{2}+2H_{2}O\to Ca{SO}_{4}\cdot 2H_{2}O+2NaOH$$

(3) $${Na}_{2}{SO}_{4}+3CaO\cdot 2SiO_{2}\cdot 3H_{2}O+H_{2}O\to Ca{SO}_{4}\cdot 2H_{2}O+2NaOH+SiO_{2}\cdot H_{2}O$$

(4) $$3\left(Ca{SO}_{4}\cdot 2H_{2}O\right)+3CaO\cdot {Al}_{2}O_{3}\;12H_{2}O+14H_{2}O\to 3CaO\cdot {Al}_{2}O_{3}\;3Ca{SO}_{4}\cdot 12H_{2}O$$

The type of crystal produced by the reaction between the sodium sulfate solution and the products of hydration depends on its concentration. Ettringite crystals are formed when the concentration of sulfate ions in the solution is lower than 1000 ppm, and gypsum crystals are formed when their concentration is higher than 8000 ppm [37]. The higher the concentration of the sulfate ions, the greater the susceptibility of concrete to erosion. The degree of erosion of concrete can be divided into slight erosion (${SO}_{4}^{2-}<$ 150 ppm), moderate erosion (150 ${<SO}_{4}^{2-}<$ 1500 ppm), severe erosion (1500 ${<SO}_{4}^{2-}<10,000$ ppm), and very severe erosion (${SO}_{4}^{2-}>$ 10,000 ppm) according to the concentration of sulfate ions. We used a sodium sulfate solution with a concentration of 5% in the test, and it had a very severe erosive effect while producing gypsum crystals.

#### 3.3.2. Crystallization Pressure

**Figure 13:**
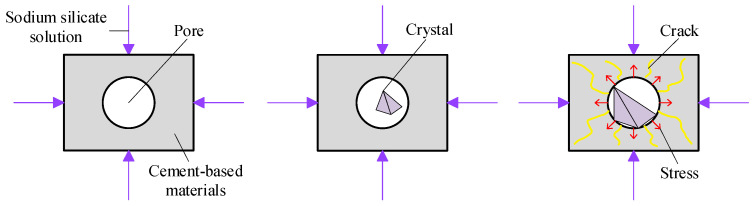
Strain and crack development mechanism.

Correns [38] experimentally demonstrated the existence of the crystallization pressure in 1949 and quantified it using thermodynamic equations. The so-called crystallization pressure is the force of interaction between the solid product formed by the chemical reaction between sulfate and cement stone. Sodium sulfate crystallizes from water to form mirabilite; its volume expands by four to five times, and the expansion force causes concrete to crack such that its performance deteriorates. Sodium sulfate reacts with the products of hydration to form gypsum crystals, where this increases the volume of concrete by 1.2 times, and the resulting swelling force causes it to crack. Sodium sulfate undergoes a chemical reaction to form crystals, which grow and swell. The expansion force causes tensile stress along the edge of the concrete pores. When the tensile stress exceeds the tensile strength of concrete, cracks are generated, and the complex process of the sodium sulfate solution eroding concrete commences. Specifically, Figure 13 shows that when sulfate ions diffuse through the concrete material, they react with the products of hydration in the cement stone, and the products of this reaction are evenly distributed. If the volume of the product is greater than the volume of the reactant, the excess volume can be accommodated only in the pores of the cement stone. The crystals in the pores are deposits of excess solid. As the reaction progresses, the volume of the solid sediments slowly accumulates. When they come into contact with the wall of the hole, the resulting pressure leads to stress in the material. When this stress exceeds the tensile strength of concrete, nanocracks begin to form in it. When the solid sediment does not have the same shape as the pores, it does not fill them to generate pressure.

The gypsum crystals in concrete are produced by the joint reactions involving calcium ions in the products of hydration and sodium ions in the sodium sulfate solution and the aqueous solution. The formula is as follows:

(5) $${Ca}^{2+}+{SO}_{4}^{2-}+2H_{2}O\to Ca{SO}_{4}\cdot 2H_{2}O$$

The reaction rate is as follows:

(6) $$\gamma_{G}=\frac{1}{V_{p}}\frac{d\varepsilon }{dt},$$

where $\gamma_{G}$ is the chemical reaction rate, mol/Ls; $V_{p}$ is the volume of sodium sulfate penetrating into the pores of the concrete, L; ε is the reaction progress, mol; and t is the reaction time, s.

According to the quantification of the mass effect, when the reaction time is from time 0 to time i, it can be obtained as follows:

(7) $$\gamma_{G}=k_{G}\cdot c_{{Ca}^{2+}}\cdot c_{{SO}_{4}^{2-}}$$

where $k_{G}$ is the constant of the chemical reaction rate of the gypsum crystal, $c_{{Ca}^{2+}}$ is the concentration of calcium ions, mol/L, and $c_{{SO}_{4}^{2-}}$ is the concentration of sulfate ions, mol/L.

Combine Equation (7) to Equation (6) integrals as follows:

(8) $$\varepsilon =k_{G}\cdot c_{{Ca}^{2+}}\cdot c_{{SO}_{4}^{2-}}\cdot V_{p}\cdot t_{i}$$

The number of gypsum crystal particles produced by the reaction is as follows:

(9) $$N_{G}=N_{G}^{0}+\varepsilon$$

where $N_{G}$ is the number of gypsum crystal particles, mol, and $N_{G}^{0}$ is the number of gypsum crystal particles at 0 time, mol.

Combine Equations (8) and (9) as follows:

(10) $$N_{G}=N_{G}^{0}+k_{G}\cdot c_{{Ca}^{2+}}\cdot c_{{SO}_{4}^{2-}}\cdot V_{p}\cdot t_{i}$$

Because no chemical reaction has occurred at time 0, $N_{G}^{0}$ is 0. Therefore, the number of gypsum crystal particles formed by the reaction at time i is as follows:

(11) $$N_{G}=k_{G}\cdot c_{{Ca}^{2+}}\cdot c_{{SO}_{4}^{2-}}\cdot V_{p}\cdot t_{i}$$

Suppose the time for which the specimen is soaked in the sodium sulfate solution in each W–D cycle is $t_{i}$. In this case, the concentration of the sodium sulfate solution in the pores of concrete increases with the number of cycles, and the tolerance is the initial concentration of the sodium sulfate solution. Assuming that the solution becomes saturated after *N* cycles, the number of particles of gypsum generated in the pores of concrete after *N* cycles is as follows:

(12) $$N_{G}^{N}=\frac{N\left(N+1\right)}{2}k_{G}\cdot c_{{Ca}^{2+}}\cdot c_{{SO}_{4}^{2-}}^{0}\cdot V_{p}\cdot t_{i}$$

where $N_{G}^{N}$ is the number of gypsum crystal particles in the pores after *N* cycles, mol, and $c_{{SO}_{4}^{2-}}^{0}$ is the initial concentration of sodium sulfate solution, mol/L.

The volume of gypsum crystals formed after *N* cycles is as follows:

(13) $$V_{G}^{N}=\frac{N\left(N+1\right)}{2}k_{G}\cdot c_{{Ca}^{2+}}\cdot c_{{SO}_{4}^{2-}}^{0}\cdot V_{p}\cdot t_{i}\cdot v_{G}$$

where $V_{G}^{N}$ is the volume of gypsum crystals formed after *N* cycles, L, and $v_{G}$ is the molar volume of gypsum crystals, L/mol.

If the sodium sulfate solution continues to undergo W–D cycles after reaching saturation, its concentration in the pores remains constant. We carried out *M* cycles after the solution had become saturated, and the number of crystal particles of gypsum formed in the pores of concrete is as follows:

(14) $$N_{G}^{M}=M\cdot k_{G}\cdot c_{{Ca}^{2+}}\cdot c_{{SO}_{4}^{2-}}^{sat}\cdot V_{p}\cdot t_{i}$$

where $N_{G}^{M}$ is the number of gypsum crystal particles in the pores after *M* cycles, mol, and $c_{{SO}_{4}^{2-}}^{sat}$ is the saturation concentration of sodium sulfate solution, mol/L.

The volume of gypsum crystals formed after *M* cycles is as follows:

(15) $$V_{G}^{M}=M\cdot k_{G}\cdot c_{{Ca}^{2+}}\cdot c_{{SO}_{4}^{2-}}^{0}\cdot V_{p}\cdot t_{i}\cdot v_{G}$$

where $V_{G}^{M}$ is the volume of gypsum crystals formed after *M* cycles, L.

We used sodium sulfate with a very high concentration of 5% in the W–D cycle tests conducted here. The crystallization pressure in the pores of concrete was due mainly to the gypsum crystals. The crystallization pressure of gypsum crystals can be calculated as follows [39,40]:

(16) $$P=\frac{RT}{V_{s}}ln\left(\frac{Q_{reac}}{K_{reac}}\right)$$

(17) $$Q_{reac}=\prod_{e}{a_{e}}^{v_{e}^{reac}}$$

(18) $$K_{reac}=exp\frac{\mu_{G}^{\Theta }-\mu_{{Ca}^{2+}}^{\Theta }-\mu_{{SO}_{4}^{2-}}^{\Theta }}{RT}$$

where $Q_{reac}$ is the product of ion activity, $K_{reac}$ is the equilibrium constant of the chemical reaction, $a_{e}$ is the activity of ion e in solution, $v_{e}^{reac}$ is the stoichiometric coefficient, $\mu_{G}^{\Theta }$ is the standard chemical potential of gypsum crystals, $\mu_{{Ca}^{2+}}^{\Theta }$ is the standard chemical potential of gypsum crystals, and $\mu_{{SO}_{4}^{2-}}^{\Theta }$ is the standard chemical potential of gypsum crystals. When the temperature is 25 °C and the pressure is 101.325 kPa, $K_{reac}$ = 2.627 × 10^−5^.

#### 3.3.3. Structural Damage

**Figure 14:**
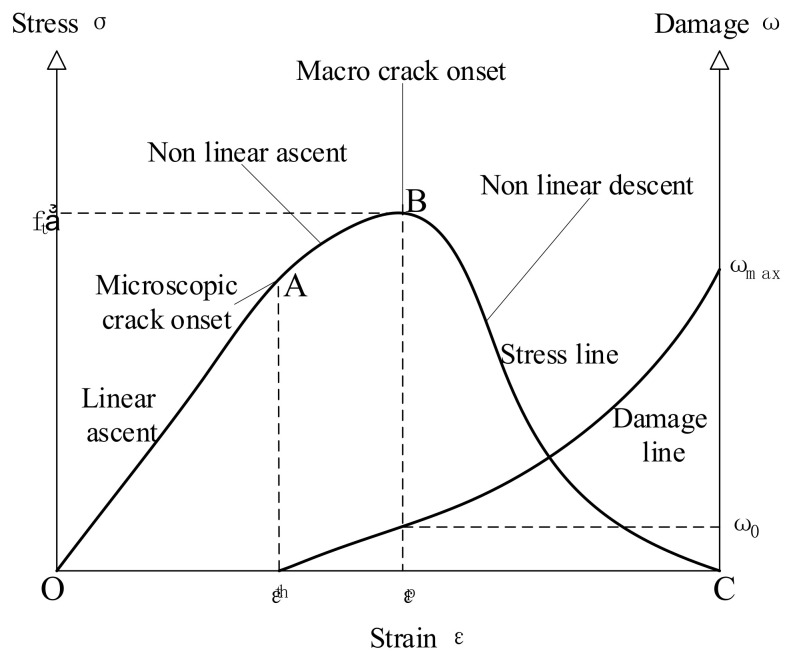
Stress–strain diagram of concrete under tension [13].

Material damage refers to the process of deterioration of the mechanical properties of the microstructure of the material under an external load or environmental action. It is also the process of the initiation, development, and polymerization of microdefects. The expansion and deformation of concrete materials increase with the stress caused by the crystallization pressure in their pores such that the density of cracks in them increases, and the damage continues to dynamically develop from their surface to inside them. The changes in damage to the concrete material with stress and strain are shown in Figure 14. Section OA represents the elastic stage of concrete, in which it contains pores and microcracks, but this does not affect its strength such that it sustains no damage. Section AB is the stage of a non-linear increase, in which new microcracks begin to form at point A, while the microcracks at point B gather to form macroscopic cracks that lead to the failure of concrete. Section BC represents the stage of a non-linear decline because the concrete has been damaged. Its stress decreases rapidly, the strain on it continues to increase, and the material enters the softening stage until the structure fails. Damage to concrete material reaches its maximum value at this time. In the relevant figure, f_t_’ is the peak tensile stress, ε^th^ is the threshold strain at which microcracks form, ε^p^ is the strain corresponding to the peak stress, ω_0_ is the damage corresponding to the peak stress, and ω_max_ is the maximum damage.

To explore the damage to concrete under the W–D cycles, we introduce the concept of the rate of reduction in the velocity of ultrasonic waves. This is calculated as follows:

(19) $$P_{N}=\frac{V_{0}-V_{N}}{V_{0}}$$

where $P_{N}$ is the rate of ultrasonic wave velocity attenuation after *N* W–D cycles, %; $V_{0}$ is the ultrasonic wave velocity before the W–D cycles, m/s; and $V_{N}$ is the ultrasonic wave velocity after *N* wet and dry cycles, m/s.

**Figure 15:**
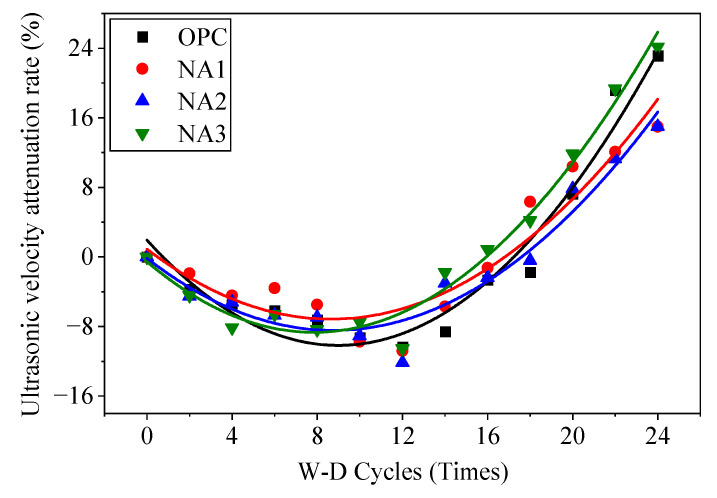
Ultrasonic velocity attenuation fitting curve.

According to the law of reduction in the velocity of ultrasonic waves in the concrete samples, we found that the relation between the rate of reduction in their velocity and the number of W–D cycles was an inverted parabolic curve. We thus used the quadratic function $P_{N}=aN^{2}+bN+c$ for a regression analysis of the data. The dots in Figure 15 represent the test data, and the lines are the curves of the fitting. We obtained a model of ultrasonic damage in different specimens by fitting the curves, as shown in Table 4. The model of damage was used as a reference model to analyze the decline in the performance of concrete materials during erosion by the sodium sulfate solution in the W–D cycles.

**Figure 16:**
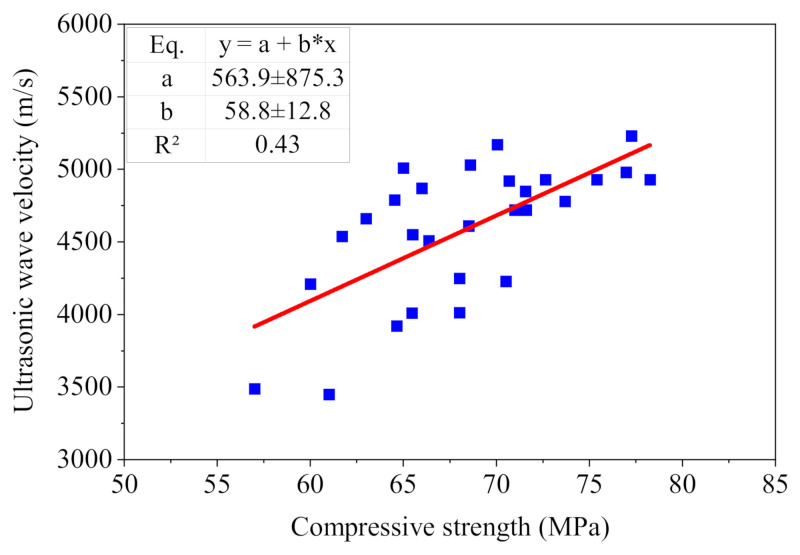
Relationship between ultrasonic wave velocity and compressive strength.

The results of the W–D cycle test showed that the compressive strength and velocity of ultrasonic waves of the concrete sample increased first and then decreased with an increasing number of W–D cycles. We thus analyzed the correlation between the compressive strength and the velocity of ultrasonic waves of the concrete samples. The equation of correlation and the relational coefficient (R^2^ = 0.43) were obtained, as shown in Figure 16. The results show that the correlation between the compressive strength and the velocity of ultrasonic waves in it was very low. We thus recommend using multiple indicators to comprehensively evaluate damage to concrete materials.

## 4. Conclusions

Nano-alumina has a significant impact on the performance of concrete owing to its unique physical and chemical properties. In this study, we examined the macroscopic characteristics, microstructure, and mechanism of damage of NA-modified concrete subjected to erosion by a sodium sulfate solution. The following conclusions were obtained:

(1) NA-modified concrete had a higher resistance to erosion by the sodium sulfate solution than ordinary concrete; specimen NA1 delivered the best performance of all samples considered, and, therefore, it is recommended to use NA1 as nano-additives in concrete.
(2) The erosion damage of sodium sulfate is mainly reflected in the internal structure of concrete, such as the increase in the proportion of harmful pores, which leads to the deterioration of the compactness of concrete so that the durability is greatly reduced.
(3) Erosion by sodium sulfate was caused by crystals produced by the chemical reaction with mainly ettringite and gypsum crystals. They were deposited in the pores of concrete to generate crystallization pressure. When this pressure exceeded the tensile strength of concrete, cracks were formed in it, and the process of damage to it was initiated.
(4) The concrete damage model based on ultrasonic velocity can be used to evaluate the degree of damage to concrete caused by sulfate erosion.

## Figures and Tables

**Table 1:** Chemical component of cement.

Chemical Composition and Mass Ratio (%)								
CaO	SiO_2_	Al_2_O_3_	Fe_2_O_3_	MgO	K_2_O	TiO_2_	SO_3_	Loss
69.89	17.30	3.14	3.76	2.09	0.64	0.18	2.25	2.78

**Table 2:** Physical properties of nano-Al_2_O_3_.

**Physical properties**	Diameter	Bulk density	Specific surface area	Purity
	20 nm	0.25 g/cm^3^	153 m^2^/g	99.9%

**Table 3:** Mix proportion of concrete (kg/m^3^).

Sample	W/B	Cement	NA	Water Reducer	Sand	Aggregate
OPC	0.4	440.0	0.0	4.4	678.0	1106.0
NA1	0.4	435.6	4.4	4.4	678.0	1106.0
NA2	0.4	431.2	8.8	4.4	678.0	1106.0
NA3	0.4	426.8	13.2	4.4	678.0	1106.0

**Table 4:** Ultrasonic wave velocity attenuation model.

Sample	Function	a	b	c	R^2^
OPC	P_N_ = aN^2^ + bN + c	0.1496	−2.6893	1.9219	0.9501
NA1		0.1073	−1.8579	0.8863	0.9136
NA2		0.1081	−1.8923	−0.1676	0.9347
NA3		0.1322	−2.0721	−0.5507	0.9728

## Data Availability

No data, models, or code were generated or used during the study.
